# Complete plastome sequence of *Vernicia Montana* Lour. (Euphorbiaceae): a deciduous tree species in southeast Asia

**DOI:** 10.1080/23802359.2021.1899864

**Published:** 2021-03-18

**Authors:** Jian-Feng Yao, Xiao-Feng Zhang, Zhi-Xin Zhu, Hua-Feng Wang

**Affiliations:** Hainan Key Laboratory for Sustainable Utilization of Tropical Bioresources, College of Tropical Crops, Hainan University, Haikou, China

**Keywords:** *Vernicia montana* Lour, Euphorbiaceae, Plastome, Genome structure, Phylogenomics

## Abstract

*Vernicia montana* Lour. is a deciduous tree species belonging to the family of Euphorbiaceae, distributed in southeast Asia. Here, we report and characterize the complete plastome of *Vernicia montana* Lour. The complete plastome is of 164,506 bp in length with a typical structure and gene content of angiosperm plastome, including two inverted repeat (IRs) regions of 27,965 bp, a large single-copy (LSC) region of 91,427 bp and a small single-copy (SSC) region of 17,149 bp. The plastome contains 130 genes, consisting of 81 protein-coding genes (six of which are repetitive in IR), 38 tRNA genes (seven of which are repetitive in IR), seven rRNA genes (5S rRNA, 4.5S rRNA, 23S rRNA and 16S rRNA) (three of which are repetitive in the IR), and four pseudogenes. The overall G/C content in the plastome of *Vernicia montana* Lour. is 35.8%. The complete plastome sequence of *montana* Lour. will provide a useful resource for the conservation genetics of this species as well as for phylogenetic studies in Euphorbiaceae.

## Introduction

*Vernicia montana* Lour. (Euphorbiaceae) is a deciduous tree species in the family of Euphorbiaceae, up to 20 meters in height. It is distributed in Central and south China, also distributed in Myanmar, Thailand and Vietnam. It is also cultivated in Japan. It grows in the open forests below 1600 m above the sea level. The seeds of *Vernicia montana* Lour. are a source of drying oils, could be used in paints and varnishes (Li et al. 2008; Zhou et al. 2020) The complete plastome information and the phylogeny of *Vernicia montana* Lour. with its closer relatives have not been reported. We reported and described the complete plastome of *Vernicia montana* Lour. in this study, its GenBank accession number is MW297080, which would be beneficial to promote collection, preservation and phylogenetic study of Euphorbiaceae and its related taxa.

In this study, *Vernicia Montana* Lour. was sampled from Ruili county of Yunnan province, China (97.84°E, 24.00°N). A voucher specimen (voucher code: RL0876, Wang et al., GPSII-001) and its DNA was deposited in the Herbarium of the Institute of Herbarium of China National GeneBank (code of herbarium: HCNGB).

The experiment was carried out as reported in Zhu et al. (2018). With MITO bim v1.8 (le-petit-quevilly, France) (Hahn et al. 2013), about six Gb of cleaning data was assembled for the plastid group of *Jatropha curcas* NC012224.1 (Rivarola et al. 2011). Using Geneious R8.0.2 (Biomatters Ltd., Auckland, New Zealand), the plastome was annotated against the plastome of *Vernicia fordii* (Hemsl.) Airy Shaw, NC034803.1. The annotation was corrected with CPGAVAS2 and Geseq (Tillich et al. 2017; Shi et al. 2019).

The whole length of the plastome of *Vernicia montana* Lour. possesses 164,506 bp with the typical quadripartite structure of angiosperms, contains two Inverted Repeats (IRs) of 27,965 bp, a Large Single-Copy (LSC) region of 91,427 bp and a small single-copy (SSC) region of 17,149 bp. The plastome contains 130 genes, consisting of 81 protein-coding genes (six of which are duplicated in the IR), 38 tRNA genes (seven of which are duplicated in the IR) and seven rRNA genes (5S rRNA, 4.5S rRNA, 23S rRNA and 16S rRNA) (three of which are duplicated in the IR). Among these genes, there are four pseudogenes. The overall G/C content in the plastome of *Vernicia montana* Lour. is 35.8%, which the corresponding value of the LSC, SSC and IR region were 33.3%, 30.0% and 41.8%, respectively.

**Figure 1.**
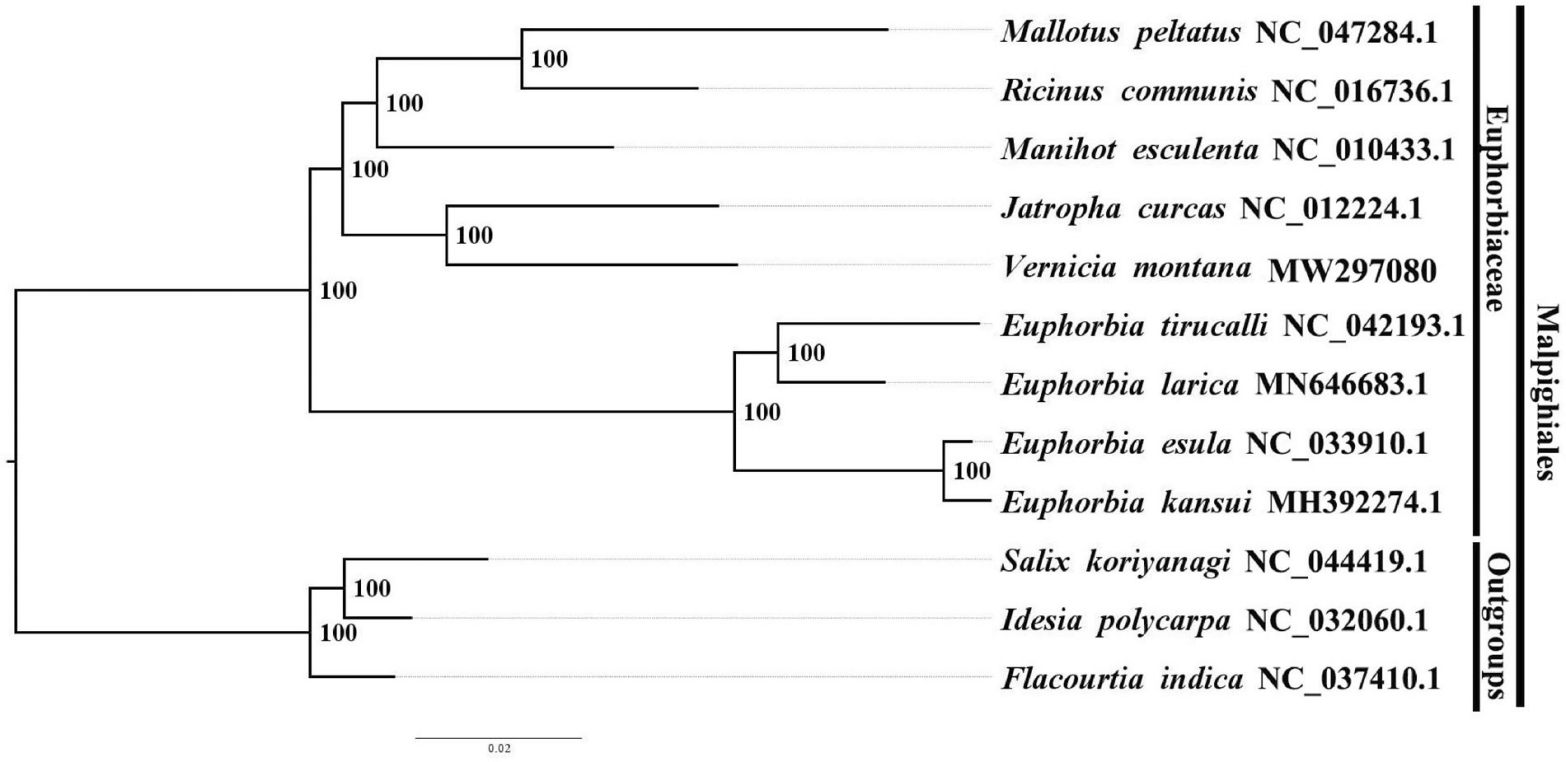
The maximum likelihood (ML) phylogeny recovered from 12 complete plastome sequences by RAxML.

We used RAxML (Stamatakis 2006) with 1000 bootstraps under the GTRGAMMAI substitution model to reconstruct a maximum likelihood (ML) phylogeny of eight published complete plastomes of Euphorbiaceae, using *Salix koriyanagi* Kimura ex Gorz, NC044419.1, *Idesia polycarpa* Maxim., NC032060.1 *and Flacourtia indica* (Burm. f.) Merr., NC037410.1 as outgroups. By constructing phylogenetic relationship, we find that *Vernicia montana* Lour. is closer to *Jatropha curcas* Linn. than other members within Euphorbiaceae in this study (Figure 1). Most nodes in the plastome ML tree were highly supported. The complete plastome sequence of *Vernicia Montana* Lour. can be better carried out the resource development and protection project of Euphorbiaceae plants, making a better understanding on the phylogeny of Euphorbiaceae plants.

## Data Availability

The genome sequence data that support the findings of this study are openly available in GenBank of NCBI at (https://www.ncbi.nlm.nih.gov/) under the accession no. MW297080. The associated BioProject, SRA, and Bio-Sample numbers are PRJNA438407, SRS3261036 and SAMN08770958, respectively.
